# Comparison of outcomes between conventional lumbar fenestration discectomy and minimally invasive lumbar discectomy: an observational study with a minimum 2-year follow-up

**DOI:** 10.1186/1749-799X-8-34

**Published:** 2013-09-24

**Authors:** Shiju A Majeed, C S Vikraman, Vivek Mathew, Anish T S

**Affiliations:** 1 Department of Orthopedics, Government Medical College, Trivandrum, India; 2 Department of Community Medicine, Government Medical College, Trivandrum 695011, India

**Keywords:** Fenestration discectomy, Minimally invasive lumbar discectomy, MLD

## Abstract

**Background:**

Different surgical techniques for lumbar discectomy are in vogue. This study compares the outcomes of two techniques for lumbar discectomy, *viz.* micro lumbar discectomy (LD) and conventional fenestration discectomy.

**Materials and methods:**

Sixty-six patients who had single-level 'virgin’ lumbar disc herniation with unilateral radicular symptoms were included. Of these, 39 had undergone MLD while 27 had undergone fenestration. Outcomes were measured using the visual analogue scale (VAS) for back and leg pain, Japanese Orthopedic Association (JOA) score, Roland-Morris score (RM) improvement and North American Spine Society (NASS) score. All quantitative data were summarised using mean and standard deviation, and qualitative data using proportions. Significance of differences across the two groups in terms of mean scores was assessed using independent sample *t* test, and the improvement within the same groups was measured using paired t test. Multiple linear regression analysis was done to assess independent predictors of improvement.

**Results:**

The MLD group showed statistically better outcomes with regard to improvement in JOA score at 6 weeks, 6 months and 2 years. Mean (SD) VAS for lower back ache at 6 weeks, 6 months, and 2 years was better for the MLD group. But the difference noted in VAS for leg pain was not statistically significant across the groups (*P* = 0.133). The improvement noted in JOA at 2 years postoperatively compared to the preoperative score was 13.67 (2.89) in the MLD group and 12.11 (3.30) in the macrodiscectomy group (*P* = 0.046).

The mean (SD) RM improvement for the MLD group was 79.24% (8.96%) vs 71.72% (16.53), *P* = 0.02, in the macrodiscectomy group. Mean NASS score for the MLD group was 2.74 vs 2.96 in the conventional group (*P* = 0.407).

The type of surgery was the significant predictor of improvement in JOA score (*P* = 0.046) even after adjusting for age, sex, level of lesion and the initial JOA score. MLD as the surgical procedure (*P* = 0.002) and a lower initial JOA score (*P* = 0.006) were found significantly contributing to the RM improvement.

**Conclusion:**

The study shows that both MLD and fenestration give comparable results at short-term follow-up. There is statistically significant improvement in MLD with regard to improvement in JOA, VAS and RM scores at 2 years. However, the difference is not large and may not be clinically significant.

## Background and introduction

Discectomy for symptomatic lumbar disc herniation is a commonly performed spinal surgical procedure. Mixter and Barr performed the first lumbar discectomy by a laminectomy and transdural approach in 1934. Semmes described the hemilaminectomy approach with retraction of the dura to remove the disc. Discectomy via a laminectomy was the popular approach for a long time. However, this involved removal of a large amount of normal bone, muscle tissue and sometimes facet joints which resulted in iatrogenic instabilities to the spine and failed back syndromes. Hence, conventional laminectomy and discectomy has been replaced by bone-sparing techniques. With the advent of better retractor systems and illumination and magnification, discectomies are performed via a more conservative route of interlaminar approaches. Surprisingly, Lowe [1] described his interlaminar fenestration technique as early as 1939. Surgeons have modified Lowe’s technique to make it more tissue sparing. Conventional fenestration technique used bilateral paraspinal muscular elevation and larger incisions and retractor systems. Interlaminar approach was used to enter the epidural space. Minimally invasive techniques evolved where paraspinal muscular elevation is done for only 2 to 3 cm using specialised retractor systems. Caspar [2] in 1977 and Williams [3] in 1978 described microlumbar discectomy technique. Adequate illumination and magnification are achieved via the use of microscopes, operating loupes and head lamps or endoscopes. Minimally invasive techniques have the theoretical advantage of less tissue scarring and better visualisation of the dura, roots and disc space (as they are done under magnification of operating loupes or microscopes), and hence are expected to have better postoperative outcomes.

We attempted to compare outcomes of two techniques for lumbar discectomy, *viz.* conventional open fenestration and minimally invasive lumbar discectomy (MLD). The procedures were performed in a tertiary care teaching hospital.

## Materials and methods

The study was conducted after approval from the Human Ethics Committee of the Government Medical College, Trivandrum, after institutional research board clearance. Case records of patients who had undergone lumbar discectomy in our unit for lumbar disc herniation from 2005 to 2008 were analysed. Patients with single-level 'virgin’ lumbar disc herniations producing unilateral lumbar radiculopathy were selected for the study. Patients with stenosis, bilateral involvement, multiple disc herniations, revision surgeries and cauda equina syndrome were excluded. All patients had undergone MRI scanning of the spine. Six weeks of conservative care was given to all patients. Clearance from the institutional review board was obtained for the study. Out of a total of 125 discectomy procedures done, 75 patients matched the above criteria. Preoperative Japanese Orthopedic Association (JOA) [4] scoring and visual analogue scale (VAS) scoring for lower back ache (LBA) and radicular leg pain were done in all patients. Among these 75 patients, we identified two sets of patients: one group (group A, *n* = 43) who had undergone minimally invasive discectomy and another group (group B, *n* = 32) who had undergone conventional fenestration type of lumbar discectomy. In group A, four patients could not be followed up for 2 years, and in group B, five were lost to follow-up. That makes 39 patients in group A and 27 patients in group B. The choice of a particular procedure was solely dependent on the operating surgeon. Two surgeons (CSV and SM) performed the procedures.

Conventional fenestration discectomy involved longer incisions (average of 7 cm), bilateral paraspinal muscle elevations, laminotomy/flavotomy and discectomy. The levels were identified by exposing the first sacral vertebra and counting upwards. In minimally invasive lumbar discectomies, the operating level was first identified by putting a marker overlying the disc space, and a C-arm image was taken. Skin incision of 3.1-cm average was centred on the marker. The paraspinal elevation was done only on the symptomatic side. Cases were operated with the help of an operating loupe or microscope. Specialised retractors were used for this type of surgery.

Patients were analysed on the basis of preoperative JOA score, VAS for low back pain and leg pain, operative time, operative blood loss, postoperative JOA score, postoperative VAS for back pain and leg pain, Roland-Morris [5] score and North American Spine Society (NASS) [6] score. The postoperative assessments were done at 6 weeks, 6 months and 2 years.

## Results

All quantitative data were summarised using mean and standard deviation, and qualitative data using proportions. Significance of differences across the two groups in terms of mean scores was assessed using independent sample *t* test, and the improvement in score of the same groups were measured using paired *t* test. The independent predictors of the differences in improvement were assessed by multiple linear regressions. A significance level of 95% and a power of 80% have been fixed for all the analyses.

A total of 66 individuals were studied. The mean (standard deviation) of age distribution was 37.45 years (8.69). The youngest in the group was 22 years and the oldest was 60 years. Out of the total, 21 (31.8%) were women. Forty-two (63.6%) patients had a lesion at the L4–L5 level, and the rest (24, 36.4%) had a lesion at the L5–S1 level. Minimally invasive lumbar discectomy was the procedure opted in the case of 39 (59.1%) patients, and conventional fenestration was done in 27 (40.9%) patients. The various clinical/perceived pain scores at the time of admission are given in Table 1.

**Table 1:** The preoperative JOA and VAS scores

**Preoperative scores**	**Group A, mean (SD)**	**Group B, mean (SD)**	***P *value (*t *test)**
JOA	11.26 (2.22)	10.19 (1.82)	0.043^a^
VAS for LBA	4.81 (1.92)	7.33 (0.68)	<0.001^a^
VAS for leg pain	7.23 (1.38)	7.70 (0.67)	0.104

Group A, MLD group; Group B, conventional. ^a^Difference found is significant.

The time taken (mean (SD)) for surgery in the case of microlumbar discectomy (60.74 min (16.56)) and that for macrodiscectomy (64.81 min (19.44)) were not different statistically (*P* value = 0.364). However, the blood loss during the procedure was significantly less with a mean (SD) of 68.72 ml (27.28) in the case of minimally invasive lumbar discectomy compared to 212.96 ml (40.65) in the case of macrodiscectomy, *P* value < 0.001.

A significant reduction was noted in perceived pain score (visual analogue score) for LBA and for leg pain in both the study groups at 6 weeks postoperatively (*P* < 0.001). The minimally invasive (micro)lumbar discectomy group had a mean (SD) visual analogue score for low back ache of 2.28 (1.12) and the fenestration discectomy group had a score of 3.17 (1.02). The difference was found to be significant statistically (*P* = 0.002). But the difference noted in the case of visual analogue score for leg pain was not statistically significant across the groups (*P* = 0.133), with a mean (SD) score of 2.31 (1.24) in the minimally invasive lumbar discectomy group and 2.76 (1.10) in the macro(fenestration) discectomy group.

A further reduction was noted in perceived pain score (visual analogue score) for LBA and for leg pain in both study groups at 6 months postoperatively (*P* < 0.001). The minimally invasive lumbar discectomy group had a mean (SD) visual analogue score for low back ache of 1.28 (0.97) and the macrodiscectomy group had a score of 2.89 (1.48). The difference was found to be significant statistically (*P* < 0.001). The difference noted in the case of visual analogue score for leg pain was not statistically significant - minimally invasive lumbar discectomy group (*P* = 0.133) had a mean (SD) score of 0.77 (1.20) compared to 2.44 (1.63) in the macrodiscectomy group. The various clinical/perceived pain scores at 2 years after the surgery are given in Table 2.

**Table 2:** The JOA scores and VAS score at 2 years postoperatively

	**MLD group**	**Conventional group**	***P *value ( *t *test)**
JOA	24.92 (2.61)	22.30 (2.40)	0.046^a^
VAS for LBA	0.90 (0.94)	2.89 (1.48)	<0.002^a^
VAS for leg pain	0.77 (1.00)	2.44 (1.63)	<0.133
NASS score	2.74 (1.16)	2.96 (0.85)	0.407
RM % improvement	79.24 (8.96)	71.72 (16.53)	0.020^a^

^a^Difference found is significant.

The improvement noted in JOA over the time of 2years post surgery compared to that of the initial score was at an average of (mean(SD)) 13.67 (2.89) in the case of minimally invasive lumbar discectomy and 12.11 (3.30) in the case of macrodiscectomy. The minimally invasive lumbar discectomy group had achieved a significantly higher rate of improvement (*P* = 0.046) in spite of the high initial score (Table 1). Comparing postoperative JOA at 2 years with preoperative JOA reflects better functional status of the patients than JOA scoring done at 6 weeks and 6 months.

Multiple linear regression analysis was performed to find out the independent predictors of reduction in JOA score. The type of surgery was the significant predictor of improvement (*P* = 0.046) even after adjusting for age, sex, level of lesion and the initial JOA score. The same analysis was done keeping Roland-Morris score (RM) improvement as the outcome variable, and two factors, minimally invasive lumbar discectomy as the surgical procedure (*P* = 0.002) and a lower initial JOA score (*P* = 0.006), were found significantly contributing to RM improvement.

## Discussion

**Figure 1:**
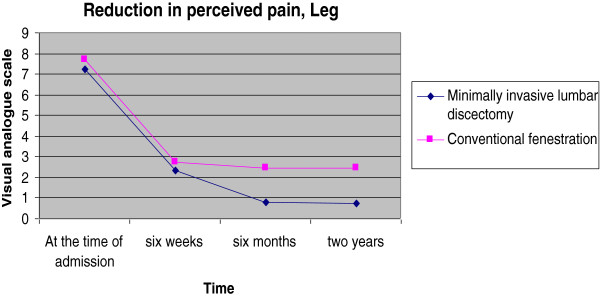
Reduction in perceived leg pain.

**Figure 2:**
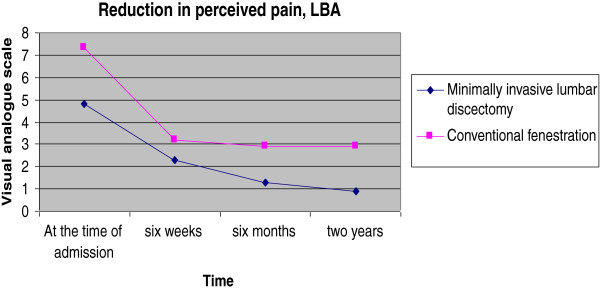
Reduction in perceived LBA.

**Figure 3:**
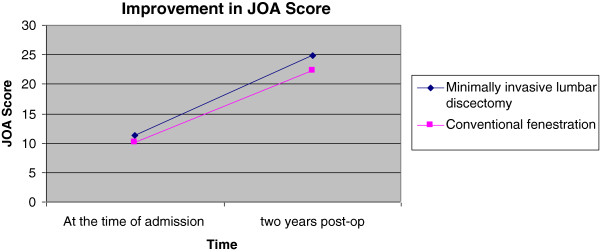
Improvement in JOA score.

Over the last decade, minimally invasive techniques have evolved as the gold standard technique in lumbar discectomy. Our study attempted to find out whether microdiscectomy has any significant advantage for the patient over the conventional fenestration surgery in a teaching hospital. Our results show that the advantage in terms of postoperative improvement is modest (Figures 1, 2 and 3). But in spite of poor initial JOA and VAS scores in the microlumbar discectomy group, the short-term outcome is better for the microdiscectomy group. The slight edge for the minimally invasive group could be due to the fact that the two groups were slightly dissimilar in the preoperative scoring as well (Table 1). However, MLD requires specialised equipment in the form of a C-arm, operating loupes or microscopes, and specialised retractors.

There are only a small number of *prospective randomised* studies comparing posterior lumbar discectomy techniques. Katayama [7] et al. studied the total volume of blood loss during surgery, comparing the conventional and microsurgical techniques, and found a statistically significant difference in favour of the microdiscectomy group. However, they concluded that there is no significant difference between the two groups in outcomes based on JOA score and VAS for leg pain. Although a statistically significant difference was noted in the duration of surgery and VAS for lumbago in the Katayama study, the difference was not large and may not be clinically significant.

Huang [8] et al. found a smaller blood loss in the group of patients treated endoscopically when compared to those treated with the classic technique. Kelly [9] et al. found that patients undergoing microdiscectomy had less tissue trauma when compared with those who underwent the classic technique; however, no difference could be noted in the clinical response. Acharya [10] et al. have reported good results in 96.5% of patients with minimally invasive lumbar discectomy in primary cases. However, there is no control group for this study. Findlay [11] et al. retrospectively reviewed a cohort of 88 patients and reported the outcome of microlumbar discectomy at 10 years. They reported an initial success rate of 91% which declined to 83% at 10-year follow-up. In a controlled randomised trial, Henrikson [12] et al. concluded that there is no significant advantage in postoperative outcomes and duration of hospital stay between conventional fenestration discectomy and microlumbar discectomy. Porchet et al. [13] in an observational study have concluded that there is no difference between the two techniques when patient response outcomes were studied. Tureyen [14] compared the outcome of single-sided, single-level, first-time lumbar disc herniation treated with and without the help of a microscope in 114 patients followed up for 1 year. They found that MLD had 90% success rate while conventional surgery had 89% success rate.

We followed the same postoperative protocol for both groups of patients. Patients were made ambulant on the third day and discharged when they felt ready to go home.

The limitation of our study is that it is not a randomised trial. Surgeon bias in choosing a particular procedure for a particular patient is a confounding factor in the study. However, the surgeons were blinded to the preoperative scoring that was used. This was done by Dr. VM (preoperative) and Dr. ATS (postoperative). Being a teaching hospital, the choice of the procedure was largely dependent on the availability of image intensifier, equipment and resources rather than on patient and image characteristics.

## Conclusion

The minimally invasive lumbar discectomy scores were only slightly better than those of the conventional discectomy in patient-rated outcomes. It can be concluded that both techniques give overall good results. Mastery of the surgeon of the procedure chosen becomes important. With regard to overall improvement in JOA score, minimally invasive lumbar discectomy is an independent predictor, irrespective of age, sex, level of lesion and initial JOA score. The theoretical advantages of MLD translating to better patient outcomes yet remain to be proven.

## Competing interests

The authors declare that they have no competing interests.

## Authors’ contributions

Dr. SM and Dr. CSV were involved in the surgical management of the patients. Dr. VM was involved in the study design, collection of data and follow-ups. Dr. ATS did the data analysis. All authors read and approved the final manuscript.
